# Analysis of maternal effect genes from maternal mRNA in eggs of *Sogatella furcifera*

**DOI:** 10.1016/j.heliyon.2024.e34014

**Published:** 2024-07-02

**Authors:** Yang Hu, Bo Feng, Fanghai Wang

**Affiliations:** State Key Laboratory for Biocontrol and Institute of Entomology, Sun Yat-sen University, Guangzhou, 510275, China

**Keywords:** Embryonic axis specification, Maternal effect gene, *Sogatella furcifera*, Transcriptome analysis

## Abstract

To understand how many kinds of mRNAs in female adults can be transferred into the eggs and the molecular basis of embryonic axis specification in *Sogatella furcifera,* we performed de novo transcriptome sequencing of six cDNA libraries of female adults and unfertilized eggs. Total 60,306 unigenes were obtained, with an average length of 1114.51 bp and N50 length of 2112 bp. Total 2900 differentially expressed genes with 496 upregulated and 2404 downregulated were found in unfertilized egg compared to female adult. Most of mRNAs in female adult could be passed into its eggs. Total 65 maternal effect genes were identified, including many homologous genes involved in embryonic axis specialization of *D. melanogaster*. This study provide transcriptome resources to elucidate the functions of maternal effect genes and the molecular basis of embryonic axis specification in *S. furcifera* in the future.

## Highlights

• Total 60,306 unigenes were obtained.
• Most of mRNAs in female adult could be passed into its eggs.
• Total 65 maternal effect genes were identified.
• Posterior polar specialization mechanisms of *S. furcifera* may be the same as that of fruit flies.
• Anterior polar specialization mechanisms are different between *S. furcifera* and fruit flies.

## 1 Introduction

Insect maternal effect genes mean that these genes are expressed in the insect mother, and then the expression product (mRNA or protein) is transported into the egg, which plays a variety of roles such as regulating or affecting the early development, later growth, development process of the embryo, and adult phenotype [1]. Early studies on fruit flies found that if the mother with the mutant gene mated with the father with the wild gene, the resulting offspring would still be mutant, thus confirming the existence of the maternal effect gene. Further studies have shown that maternal effect genes are very important for early embryonic development and can regulate the differentiation of anterior/posterior and dorso-ventral polarity [2]. In recent years, with the rapid development of sequencing technology [3] and the improvement and popularization of RNAi technology [4], it has been found that maternal effect genes play an important regulatory role in the early development of embryos. It can also affect and regulate the frequency and degree of diapause occurrence, enhance the resistance to foreign invasion (especially microbial infection), and regulate the occurrence, development time, growth rate and survival status of the offspring [5].

The white-back planthopper is an important rice pest, belonging to Homoptera, Planthopper family. Although there are many researches about this pest, they mainly focus on the biological and ecological characteristics, dimorphism of wings, control methods, etc., and rarely involve the study of early embryonic development and maternal effect genes. It is known that maternal effect genes are very important for early embryonic development and can regulate the differentiation of anterior/posterior and dorso-ventral polarity [2], so the identification of maternal effector genes will be helpful to the understanding of embryonic development and axial differentiation of the white back planthopper. In this study, we attempt to sequence and analyze the maternal mRNA in the unfertilized eggs of white planthopper to determine how many kinds of mRNAs in female adults can be transferred to the eggs. At the same time, bioinformatics analysis was used to determine how many kinds of the maternal mRNAs are the product of genes that have been shown to be maternal effects in other species, and to infer the anterior/posterior pattern specification of early embryonic development compared with Drosophila.

## 2 Results

### 2.1 Sequence assembly and functional annotation

Three biological replicates for female adult samples and their unfertilized eggs samples were designed for transcriptome sequencing. Total 123,160,466 clean reads were generated for six libraries. The average Q30 value of these libraries was 95.25 %, indicating the sequencing data were effective and reliable. The mean length and N50 length of total 60,306 unigenes was 1114.51bp and 2112 bp (Table 1). 25,509 and 11,681 unigenes had homologous sequences in the Nr and Swiss-Prot protein databases, while 7,193, 22,930, 17,247, 15,748, 20,531, 28,172 and 18,592 unigenes could be classified by COG, GO, KEGG, KOG, Pfam, TrEMBL, and eggnog databases, respectively. Total 30,116 unigenes were annotated, of them 300 ≤ length<1000 and length ≥ 1000 unigenes were 10,110 and 15,770 (Table 2). There were 50,075 unignes in unfertilized eggs samples, just a little less than 54,586 unignes in female adult samples (Table 2). It suggest that most of mRNAs in female adult could be passed into its eggs.

**Table 1:** Summary statistics of female adult and unfertilized egg of *S. furcifera* transcriptome sequencing and assembly.

Reads			
Sample	Read number	Base number	%≥Q30
female adults 1	20,206,574	6,052,484,198	95.46
female adults 2	20,537,784	6,150,066,334	95.61
female adults 3	19,865,135	5,949,111,716	95.57
unfertilized eggs 1	21,016,919	6,292,718,288	95.44
unfertilized eggs 2	20,583,025	6,163,899,330	95.23
unfertilized eggs 3	20,951,029	6,273,260,106	94.20

Assembly		
Length Range	All Unignes adult Unignes	Egg Unignes
200–300	15,611 (25.86 %)	15,248 (27.93 %) 13,549 (27.06 %)
300–500	11,975 (19.84 %)	11,863 (21.73 %) 10,155 (20.28 %)
500–1000	12,677 (21.00 %)	11,936(21.87 %) 10,120 (20.21 %)
1000–2000	10,273 (17.02 %)	8636 (15.82 %) 8491 (16.96 %)
>2000	9830 (16.28 %)	6903 (12.65 %） 7760 (15.50 %)
Total number	60,306	54,586 50,075
Total length(bp)	67,278,496	52,578,366 53,424,230
Mean length (bp)	1114.51	963.22 1066.88
N50 length	2112 1759	2018
GC content	39.09 % 39.96 %	38.21

**Table 2:** Annotation result statistics between unigenes and databases.

Database	Annotation numbers	300 ≤ length<1000	length ≥ 1000
COG	7193	1975	4614
GO	22,930	7171	12,871
KEGG	17,247	4665	10,932
KOG	15,748	4175	10,155
Pfam	20,531	5843	12,920
Swissprot	11,681	2961	7723
TrEMBL	28,172	9089	15,427
eggNOG	18,592	5209	11,640
Nr	25,509	7984	14,319
All annotated	30,116	10,110	15,770

### 2.2 DEGs analysis

**Fig. 1:**
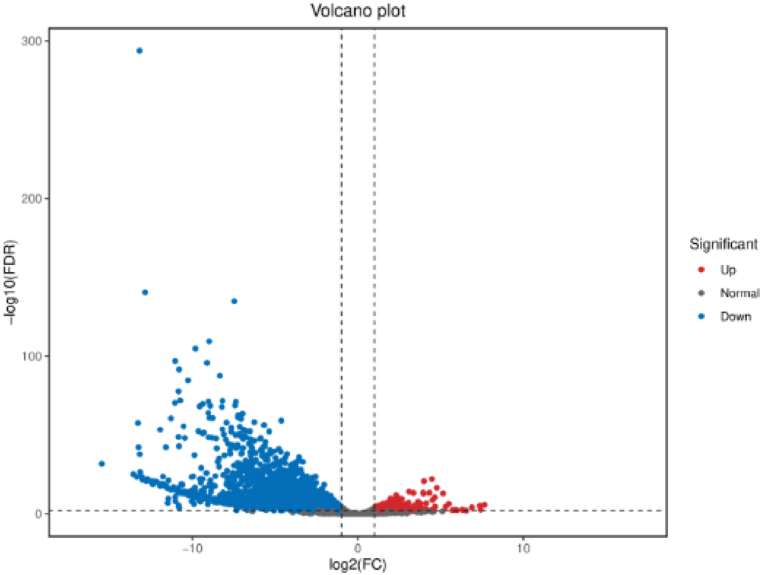
Volcano plot of DEGs between the unfertilized eggs and female adults samples. The x-axis indicates log2 FC between the two samples and the y-axis indicates the -log10 (FDR) of gene expression variation. Both red and blue dots show the significantly differential expressions (Experimental group: unfertilized egg samples, control group: female adult samples). The blue dots represent down-regulated differentially expressed genes, the red dots represent up-regulated differentially expressed genes, and the gray dots represent non-differentially expressed genes.

Total 2900 DEGs were identified. 496 DEGs exhibited relatively higher expression levels in the unfertilized eggs than the female adults, and 2404 DEGs were down-regulated in the unfertilized eggs than the female adults (Fig. 1). There were total 2128 DEGs could be annotated by COG (548), GO (1,565), KEGG (1,346), KOG (1,152), Nr (2,114), Pfam (1,548), Swiss-Prot (1,005), and eggnog (1,479) databases, respectively. The top 10 upregulated and downregulated genes in the unfertilized eggs than female adults were shown in Table 3 and Table 4.

**Table 3:** The list of top 10 up-regulated genes in unfertilized eggs than female adults.

gene ID	logFC	FDR	Annotation	Accession number	E-Value	Body-FPKM	Egg-FPKM
BMK_Unigene_25,519	6.889942468	9.18E-05	glutenin, high molecular weight subunit PW212-like	XP_022198972.1	7.30E-32	2.03	345.62
BMK_Unigene_07254	3.774959845	0.002620949	putative 3-isopropylmalate dehydratase, partial	AQT27203.1	7.10E-265	0.08	1.47
BMK_Unigene_29,791	3.727895708	0.0040893	PREDICTED: probable squalene synthase isoform X2	XP_018912292.1	4.60E-105	0.08	1.22
BMK_Unigene_23,185	3.334707731	0.000994277	tubulin	AEJ38212.1	2.70E-109	0.34	5.32
BMK_Unigene_28,133	3.065313935	8.25E-08	carboxylesterase	AWI63380.1	0	3.21	39.55
BMK_Unigene_18,992	2.77455943	8.64E-05	pancreatic triacylglycerol lipase	XP_022201851.1	1.50E-195	6.16	68.95
BMK_Unigene_02148	2.541064335	0.008408963	dual oxidase maturation factor 1-like	XP_022202371.1	5.90E-141	0.84	7.62
BMK_Unigene_35,715	2.377853979	0.001425901	putative Regulator of rDNA transcription protein 15	KAA6397628.1	4.80E-22	15.53	117.72
BMK_Unigene_19,265	2.150195327	0.000275633	leucine-rich repeat and fibronectin type-III domain-containing protein 3 isoform X1	XP_022196680.1	1.90E-153	0.28	1.85
BMK_Unigene_09131	2.102752016	0.000486872	Transposon Ty3-I Gag-Pol polyprotein, partial	KFM57554.1	1.80E-12	0.43	2.72

**Table 4:** The list of top 10 down-regulated genes in unfertilized eggs than female adults.

gene ID	logFC	FDR	Annotation	Accession number	E-Value	Body-FPKM	Egg-FPKM
BMK_Unigene_50,817	−13.29080643	2.58E-58	arrestin homolog	XP_022197820.1	9.00E-231	236.91	0.04
BMK_Unigene_00135	−13.18916202	1.63E-294	longwave opsin	BAO03858.1	7.70E-222	5807.45	0.95
BMK_Unigene_51,014	−12.85545968	2.96E-141	mucin-like protein	AQP26312.1	2.20E-266	8879.55	1.88
BMK_Unigene_01156	−12.31094679	6.31E-21	transient receptor potential-like protein	AOR81466.1	0	8.57	0
BMK_Unigene_49,246	−11.77372944	9.61E-20	shematrin-like protein 2	XP_022196647.1	6.70E-15	48.54	0.02
BMK_Unigene_38,674	−11.29369938	3.57E-61	odorant-binding protein 2	AHB59653.1	1.20E-74	641.07	0.41
BMK_Unigene_48,788	−11.16630926	4.82E-16	multicopper oxidase 4	AQS60677.1	0	11.53	0.01
BMK_Unigene_58,431	−11.10630864	7.85E-17	glycine-rich cell wall structural protein 1.8-like	XP_022186041.1	3.80E-33	42.75	0
BMK_Unigene_55,684	−10.87527591	6.59E-15	clavesin-1-like, partial	XP_022205369.1	1.70E-149	16.26	0
BMK_Unigene_52,008	−10.53440089	3.56E-56	neuropeptide-like protein 31	XP_022198836.1	1.40E-08	272.17	0.29

### 2.3 Maternal effect genes analysis

Total 65 maternal effect genes, including *tudor, staufen, pumilio, cappuccino, nudel, vasa, exuperantia,* have been identified according to embryonic axis specification (GO:0000578) and Nr notification enrichment results (Table 5). According to GO and Nr annotation, these genes may play a role in oocyte anterior/posterior axis specification, dorsal/ventral axis specification, cell projection morphogenesis, lethal maternal effect, maternal effect embryo arrest, etc. There were only five DEGs of the 65 maternal effect genes (Table 5). The five DEGs were BMK_Unigene_04802 (ras-responsive element-binding protein 1-like), BMK_Unigene_04931 (Staphylococcal nuclease domain-containing protein 1), BMK_Unigene_34,376 (polehole), BMK_Unigene_15,612 (Serine protease nudel) and BMK_Unigene_56,580 (Serine protease snake) respectively. Furthermore, FPKM of most maternal effect genes were high in the unfertilized egg samples than in the female adult samples (Table 5).

**Table 5:** The 65 maternal effect genes of *S. furcifera*.

Gene ID	Annotation	Accession number	identity%	E-value	adult FPKM	egg FPKM
BMK_Unigene_22,528	disks large 1 tumor suppressor protein	XP_022194554.1	98.24	0.00E+00	6.07	7.9
BMK_Unigene_34,370	DOM3 exoribonuclease, putative	XP_004338880.1	28.4	2.30E-10	0.04	0.65
Gene ID	Annotation	Accession number	Identity%	E-value	Adult FPKM	Egg FPKM
BMK_Unigene_22,528	disks large 1 tumor suppressor protein	XP_022194554.1	98.24	0.00E+00	6.07	7.9
BMK_Unigene_34,370	DOM3 exoribonuclease, putative	XP_004338880.1	28.4	2.30E-10	0.04	0.65
BMK_Unigene_04132	Dual specificity mitogen-activated protein kinase kinase mek-1	sp|Q21307|MEK1_CAEEL	38	3.90E-14	1.36	0.41
BMK_Unigene_13,959	Enhancer of mRNA-decapping protein 4 homolog	sp|Q9VKK1|EDC4_DROME	40.04	9.70E-83	6.59	11.71
BMK_Unigene_31,996	f-box and wd40 domain, partial (archipelago)	KMQ81669.1	65.82	6.40E-21	0.2	1.02
BMK_Unigene_12,230	F-box/WD repeat-containing protein 11	KII65815.1	42.37	1.30E-07	0	0.92
BMK_Unigene_29,299	growth factor receptor-bound protein 2	XP_022185828.1	100	5.30E-121	10.85	17.65
BMK_Unigene_59,947	heat shock protein 901	AVX33609.1	45.45	1.40E-40	4.71	0.46
BMK_Unigene_24,154	Homeotic protein female sterile	sp|P13709|FSH_DROME	43.67	8.00E-138	6.57	15.49
BMK_Unigene_07398	lethal (2) giant larvae protein	UYR00238.1	25.00 %	0.005	0.23	1.16
BMK_Unigene_32,023	Maternal protein tudor	sp|P25823|TUD_DROME	21.14	4.50E-07	8.72	18.56
BMK_Unigene_53,693	methylosome protein 50	XP_022203395.2	65.56 %	5.00E-155	5.8	4.98
BMK_Unigene_23,601	mitochondrial cardiolipin hydrolase-like	XP_020916431.1	48.82	3.30E-44	0.12	1.72
BMK_Unigene_13,947	Obscurin	sp|A8DYP0|OBSCN_DROME	37.32	8.00E-34	47.66	63.31
BMK_Unigene_33,446	Protein 60A	XP_022202841.2	96.72 %	7.00E-74	0.75	1.51
BMK_Unigene_51,702	protein gustavus	XP_022187205.1	98.4	3.60E-179	11	26.48
BMK_Unigene_28,168	Protein lines	sp|Q9V4Z9|LINES_DROME	41.58	7.50E-134	14.61	28.8
BMK_Unigene_58,178	Protein mini spindles	sp|Q9VEZ3|MSPS_DROME	45.76	0.00E+00	9.35	21.62
BMK_Unigene_04729	proto-oncogene tyrosine-protein kinase ROS-like, partial	XP_022199386.1	74.32	4.70E-68	1.17	1.1
BMK_Unigene_07984	pumilio	AGL81628.1	33.13 %	1.00E-60	0.87	1.78
BMK_Unigene_30,682	Pumilio 2	KAA0191618.1	49.47	2.20E-12	2.17	4.93
BMK_Unigene_10,378	putative RNA-binding protein 15B	XP_014231045.1	36.47 %	1.00E-07	1.05	3.46
BMK_Unigene_04802	ras-responsive element-binding protein 1-like	XP_054274568.1	52.74 %	0	2.68	12.57
BMK_Unigene_07014	Skeletor	sp|Q9VGY6|SKEL1_DROME	26.99	8.20E-52	1.84	3
BMK_Unigene_03444	small nuclear ribonucleoprotein Sm D3	XP_017961078.1	61.54	1.70E-32	1.53	2.41
BMK_Unigene_04931	Staphylococcal nuclease domain-containing protein 1	sp|Q9W0S7|SND1_DROME	27.33	5.20E-10	8.89	30.56
BMK_Unigene_28,839	Transcription factor BTF3 homolog	sp|Q18885|BTF3_CAEEL	70.86	4.10E-51	500.77	679.21
BMK_Unigene_28,886	transcription factor BTF3 homolog 4	XP_023724071.1	50.62	2.20E-30	6.67	10.52
BMK_Unigene_37,096	tropomyosin-1 isoform X2	XP_022196988.1	75.4	2.00E-85	10.99	12.2
BMK_Unigene_41,528	Vacuolar protein-sorting-associated protein 25	sp|Q55GD9|VPS25_DICDI	31.31	1.10E-08	1.17	0.44
BMK_Unigene_33,295	vacuolar protein-sorting-associated protein 25-like	XP_002156482.1	43.86	2.90E-29	0.44	1.6
BMK_Unigene_57,252	neurotrophin 1 (Spaetzle)	XP_022203620.1	80.47	7.50E-165	3.76	4.81
BMK_Unigene_26,744	trfA	sp|O77033|CYC8_DICDI	62.82	3.00E-58	1.92	3.52
BMK_Unigene_15,861	uncharacterized protein（PHD-type domain-containing protein）	XP_022188545.1	78.21	4.60E-65	0.38	1.3
BMK_Unigene_23,401	Maternal embryonic leucine zipper kinase（pig-1）	sp|U4PR86|MELK_CAEEL	33.59	6.30E-29	3.6	7.03
BMK_Unigene_15,037	MAU2 chromatid cohesion factor homolog	XP_022187847.1	98.99	0.00E+00	4.44	8.7
BMK_Unigene_41,207	mcm 3	XP_004359973.1	58.97	1.90E-16	22.78	0
BMK_Unigene_27,636	Protein maternal effect lethal 26（mel-26）	sp|Q94420|MEL26_CAEEL	25	1.10E-08	7.35	10.84
BMK_Unigene_07941	maternal effect embryo arrest 18 protein	PRP75735.1	39.64	8.20E-34	1.06	1.5
BMK_Unigene_30,014	double-stranded RNA-binding protein Staufen	AFA41503.1	96.1	0.00E+00	9.07	13.89
BMK_Unigene_23,806	Serine/threonine-protein kinase par-1	XP_022200442.1	93.65	4.70E-211	7.41	13.93
BMK_Unigene_48,124	Protein cappuccino	RZF33490.1	84.01	0.00E+00	8.5	10.49
BMK_Unigene_47,924	Protein brunelleschi	RZF46987.1	93.61	0.00E+00	5.98	8.14
BMK_Unigene_50,454	Protein mago nashi	GAV08805.1	65.13	5.60E-48	12.06	23.48
BMK_Unigene_12,748	Protein spire	XP_022193248.1	88.64	7.40E-179	7.3	13.17
BMK_Unigene_34,376	polehole	RZF33272.1	72.72	0.00E+00	2.45	8.43
BMK_Unigene_30,789	Tyrosine-protein phosphatase corkscrew	RZF32161.1	96.92	0.00E+00	4.78	6.53
BMK_Unigene_44,315	Torso-like protein	XP_022191778.1	87.57	2.00E-169	5.29	10.36
BMK_Unigene_00662	Putative transcription factor capicua	RZF39984.1	80.82	0.00E+00	2.95	5.88
BMK_Unigene_15,587	Sec23/Sec24 trunk domain	RZF39742.1	77.71	0.00E+00	9.96	18.45
BMK_Unigene_54,446	Epidermal growth factor receptor	RZF49226.1	91.04	0.00E+00	2.13	3.78
BMK_Unigene_36,828	RNA-binding protein squid	RZF46950.1	97.81	1.50E-122	9.09	21.27
BMK_Unigene_45,288	Embryonic polarity protein dorsal	AWT86616.1	100	4.00E-194	1.71	1.34
BMK_Unigene_13,133	Protein toll	RZF43732.1	91.23	1.50E-244	1.49	2.6
BMK_Unigene_15,612	Serine protease nudel	XP_022190244.1	60.07	0.00E+00	3.53	22.31
BMK_Unigene_24,491	Heparan sulfate 2-*O*-sulfotransferase pipe	XP_022187434.1	98.5	9.20E-228	3.44	7.75
BMK_Unigene_46,527	Pelle-like serine/threonine-protein kinase pik-1	RZF45921.1	81.31	2.00E-270	8.18	17.27
BMK_Unigene_56,580	Serine protease snake	RZF41610.1	79.74	2.50E-108	12.31	0.52
BMK_Unigene_11,974	Protein rhomboid	RZF49028.1	100	1.40E-166	4.63	11.28
BMK_Unigene_19,822	Serine protease gd	RZF37116.1	84.46	8.20E-223	6.6	17.24
BMK_Unigene_34,644	NF-kappa-B inhibitor cactus	RZF44463.1	76.81	8.40E-166	17.65	26.17
BMK_Unigene_57,252	Spaetzle	XP_022203620.1	80.47	7.50E-165	3.76	4.81
BMK_Unigene_32,721	protein gurken-like isoform X2	XP_022191270.1	69.81	2.90E-12	0	0
BMK_Unigene_53,947	ATP-dependent RNA helicase vasa	RZF32958.1	85.79	7.40E-285	70.27	130.72
BMK_Unigene_41,471	exuperantia (exu)	XP_022190360.1	69.38	6.70E-140	38.16	25.24

## 3 Discussion

*S. furcifera* is an important agricultural pest in china and Southeast Asian countries. It cause grievous damage to many crops, such as rice, wheat, corn and sorghum, etc. [7]. The serious threat to food production and security result from its robust fecundity and viruses transmitting, etc. [8]. Maternal effect genes play an important regulatory role in the early development of embryos, and can regulate the occurrence, development time, growth rate and survival status of the offspring [5], so they have a lot to do with pest fecundity. Although many researches on *S. furcifera* have been done, the detail maternal effect genes and embryonic axis specification mechanisms of *S. furcifera* is still unclear.

In this study, a de novo transcriptome was assembled with sequences from female adults and unfertilized eggs. A total of 2900 DEGs were identified, among which 496 DEGs exhibited relatively higher expression levels in the unfertilized eggs than the female adults (Fig. 1). Total 65 maternal effect genes have been identified according to GO and Nr notification enrichment results, these genes should have some functions involved in one or more of anterior/posterior axis specification, dorsal/ventral axis specification, cell projection morphogenesis, lethal maternal effect, maternal effect embryo arrest, etc.

The unfertilized eggs were dissected from the female adults, so all mRNAs in the unfertilized eggs were from the female adults. Hence, the up-regulated expression DEGs mean that these genes were expressed in the female adults, then mainly passed and stored in eggs. The up-regulated expression DEGs included many enzymes, such as 3-isopropylmalate dehydratase, squalene synthase, carboxylesterase, pancreatic triacylglycerol lipase, etc. (Table 3). We know egg formation and early embryogenesis is dependent on stored molecules deposited by the mother. There are many kinds of enzymes in these stored molecules, and they have been verified to play an important role in early embryogenesis of some insects [9,10].The functions of up-regulated expression DEGs in early embryogenesis of *S. furcifera* are not clear and need to be investigated in the future. The down-regulated expression DEGs mean that these genes were expressed in the female adults, and less stored in eggs. DEGs of the down-regulated expression should have some role in adult development, but less in early embryogenesis. For example, longwave-opsin showed extremely high relative ratio in adult stages of *Dendrolimus punctatus* Walker to correlate to the nocturnal lifestyles of this species at adult stage [11]; odorant-binding protein 2 was primarily expressed in the antennae of *Batocera horsfieldi* (Hope) adults to play a key role in insect olfaction [12]; clavesins are expressed exclusively in neurons to provide a unique neuron-specific regulation of late endosome/lysosome morphology in Clavesin Family [13].

Insects have evolved many mechanisms for establishing embryo polarity that are based on maternal mRNA localizations at the anterior pole or both the anterior and posterior poles of the egg [14]. Most of 65 maternal effect genes in *S. furcifera* have been found to be related to anterior/posterior axis specification or dorsal/ventral axis specification in terms of function in D. melanogaster, such as *exuperantia, staufen, vasa, pumilio, tudo, dorsal*, etc. It mean that the embryonic axis specification in *S. furcifera* somewhat similar with D. melanogaster.

There are mainly 10 genes involved in the posterior polar specialization of *Drosophila* posterior, including *polenanos*、*tudor*、*oskar*、*vasa*、*valois*、*pumilio*、*caudal*、*staufen*、*cappuccino*、*spire.* In *S. furcifera*, 8 homologous genes except *oskar and vasa* were found (Table 5), suggesting that the two insects are very similar in terms of posterior polar specialization. There are mainly 3 genes involved in the anterior polar specialization of *D. melanogaster,* including *bicoid*、*exuperantia*、*swallow.* The anterior-to-posterior Bicoid gradient polarizes this process along the primary axis by driving chromatin accessibility at *cis*-regulatory elements of target genes that need to be activated for anterior specification [15]. However, in *S. furcifera*, only *exuperantia* were found, not *bicoid* and *swallow* (Table 5), suggesting that there are some difference in terms of anterior polar specialization between *S. furcifera* and *D. melanogaster*.

In fact, among the lower dipterans (Nematocera), moth flies and culicine mosquitoes evolved new anterior determinants that are encoded by unrelated C2H2 zinc-finger genes, including *zic/odd-paired* and *cucoid*, respectively [2], not the anterior determinant gene *bicoid* from a duplicated Hox 3 ortholog in *D. melanogaster* [16]. It is interesting that we also did not find *zic/odd-paired* and *cucoid* in *S. furcifera*, suggesting that the anterior specification of *S. furcifera* is also different with the lower dipterans (Nematocera), moth flies and culicine mosquitoes. Therefor the mechanism of *S. furcifera* anterior specification will need to be further explored in the future.

## 4 Conclusion

We performed de novo transcriptome sequencing of six cDNA libraries of female adults and unfertilized eggs of *S. furcifera*. We assembled total 60,306 unigenes and annotated them by searching for homology in protein databases. Most mRNAs in female adult could be passed into its eggs. Total 2900 differentially expressed genes (DEGs) with 496 upregulated and 2404 downregulated in unfertilized egg compared to female adult, and 65 maternal effect genes were identified, including many homologous genes involved in the anterior and posterior polar specialization of *D. melanogaster*. These transcriptome data provided a fundamental support for future functional studies to elucidate the functions of maternal effect genes and the molecular basis of embryonic axis specification in *S. furcifera* and other species.

## 5 Material and methods

### 5.1 Insect culture and samples collection

Successive generations of the white-backed planthopper strain were reared on rice seedlings. The culture condition was 28 ± 2 °C with a 16:8 h light: dark cycle. The 5-instar female nymphs with long-winged bud were selected and raised in a cage alone. When they emerged into adults and grew to the point of abdominal hypertrophy (when the eggs were mature), the adults were dissected to take out the mature eggs. The eggs were washed three times using physiological saline and collected as samples of unfertilized egg, and the remaining body parts were taken as samples of female adult. Three biological replicates were set up. Each group samples of female adult included seven individuals, and the egg numbers of the three groups of unfertilized egg samples were 231, 224, and 228, respectively. All samples were snap-frozen in liquid nitrogen and stored at −80 °C prior to RNA extraction.

### 5.2 RNA isolation and sequencing

Total RNA of each sample was isolated using Trizol reagent according to manufacturer's instructions (Invitrogen, Carlsbad, CA, USA). The concentration and purity of RNA was measured using Qubit® RNA Assay Kit in Qubit®2.0 Flurometer (Life Technologies, CA, USA) and the NanoPhotometer® spectrophotometer (IMPLEN, CA, USA), respectively. The integrity of RNA was checked by electrophoresis in 1 % agarose gel and the RNA Nano 6000 Assay Kit of the Agilent Bioanalyzer 2100 system (Agilent Technologies, CA, USA). The RNA with 260/280 nm ratio between 2.0 and 2.1 was utilized to construct the cDNA library, then the qualified library was sequenced by the high-throughput sequencing platform with PE150 mode.

### 5.3 De novo transcriptome assembly and annotation

All the raw data has been submitted to NCBI Sequence Read Archive with accession numbers SRP474194 (female adult: SRR26948444, SRR26948443, SRR26948442; unfertilized egg: SRR26948441, SRR26948440, SRR26948439). The clean reads were obtain through removing the raw reads with adaptor contamination, low quality, and ambiguous base ‘N’ larger than 5 % by a custom Perl script. De novo assembly of short reads was accomplished by Trinity [6]. Functional annotations were performed by the sequence comparison of unigenes with public databases included NR, Swiss-Prot, TrEMBL, COG, KOG, eggNOG 4.5, Pfam, GO, and KEGG using the BLAST algorithm with a cutoff E value of <10^−5^, respectively.

### 5.4 Identification of differential expression genes (DEGs)

Differential expression analysis of female adult samples and unfertilized egg samples was performed using differential analysis software DESeq2. Criteria for differentially expressed genes was set as Fold Change (FC) ≥2 and False Discovery Rate (FDR) < 0.01. FC refers to the ratio of gene expression in two samples. FDR refers to the adjusted p-value used to measure the significance of the difference. In order to facilitate comparison, take the logarithmic value of the fold change as log2FC.

### 5.5 Identification of maternal effect genes

In order to authenticate genes concerned with maternal effect in *S. furcifera*, we firstly found out these genes from the GO enrichment result of all unigenes and referred to the annotation results from NR blasted with similar maternal-effect genes already reported.

## Data availability

All the raw data has been submitted to NCBI Sequence Read Archive with accession numbers SRP474194 (female adult: SRR26948444, SRR26948443, SRR26948442; unfertilized egg: SRR26948441, SRR26948440, SRR26948439).

## CRediT authorship contribution statement

**Yang Hu:** Writing – original draft, Methodology, Investigation, Data curation. **Bo Feng:** Writing – original draft, Methodology, Investigation, Data curation. **Fanghai Wang:** Writing – review & editing, Supervision, Project administration, Funding acquisition, Conceptualization.

## Declaration of competing interest

All co-authors see and agree with the contents of the manuscript, and there is no financial interest to report.
